# Neonatal Hearing Screening: protocols, obstacles and perspectives of speech therapists in Brazil - 10 years of Brazilian Federal Law 12,303/2010

**DOI:** 10.1590/2317-1782/20212020331

**Published:** 2022-01-12

**Authors:** Luíza Silva Vernier, Sílvio César Cazella, Daniela Centenaro Levandowski

**Affiliations:** 1 Programa de Pós-Graduação em Ciências da Saúde, Universidade Federal de Ciências da Saúde de Porto Alegre – UFCSPA - Porto Alegre (RS), Brasil.; 2 Departamento de Informática Biomédica, Programa de Pós-Graduação em Ciências da Saúde, Programa de Pós-Graduação em Tecnologias da Informação e Gestão em Saúde, Universidade Federal de Ciências da Saúde de Porto Alegre – UFCSPA - Porto Alegre (RS), Brasil.; 3 Departamento de Psicologia, Programa de Pós-Graduação em Ciências da Saúde, Programa de Pós-Graduação em Psicologia e Saúde, Universidade Federal de Ciências da Saúde de Porto Alegre – UFCSPA - Porto Alegre (RS), Brasil.

**Keywords:** Hearing, Cross-Sectional Studies, Public Health, Neonatal Screening, Speech, Language and Hearing Sciences

## Abstract

**Purpose:**

This study aims to know the current scenario of speech audiology therapy activities at NHS in Brazil, identifying its obstacles and perspectives, as well as verifying the adequacy of national NHS Programs to the pre-established quality indicators.

**Methods:**

Analytical observational study, carried out with speech therapists in the exercise of NHS in Brazil, between August 2018 and August 2019, through a structured online questionnaire. Descriptive and correlational analyzes of the data were performed using the SPSS version 22.0 program.

**Results:**

The effective practice of NHS was not entirely consistent with official protocols. 48.5% of speech therapists stated that NHS interruption at some point in the workplace, especially due to the need to repair the equipment (64.7%). As for the flow records and care-related data, which include quality indicators there was greater control over the total number of neonates who underwent NHS (87.9%) and less control over false-positive results (21.2%). 81.8% of speech-language-hearing therapists said they were available to use a system for recording and controlling NHS data.

**Conclusion:**

Although professionals’ practices are generally consistent with official protocols, the non-linearity of the process and the lack of data control are important obstacles to the quality of NHS services. Most of the national NHS programs presented do not meet the pre-established quality indicators. It is considered that the computerization of records can benefit professionals and enhance the implementation of NHS provided for in Brazilian laws and public policies.

## INTRODUCTION

### Brief history of newborn hearing screening in Brazil

Hearing screening tests have been used for at least 60 years to identify children who need further hearing evaluation. In the 1940s, Ewing & Ewing observed the Cochlear Palpebral Reflex^(1)^ in newborns. In 1964, Downs and Sterrit assessed the behavioral responses of neonates before a narrow-band sound stimulus centered at 3000 Hz at 90 dBNPS. In this same study, a high false-negative rate was identified and the need to create a protocol with the risk indicators for hearing impairment was identified^(2)^. Hearing screening programs specifically for newborns have been developed for over 35 years^(3)^. After the validation of the Otoacoustic Emissions (OAE) by David Kemp^(4)^, in England, the Newborn Hearing Screening (NHS) encountered a new impulse. However, approximately until the 1990s, NHS was performed in an inconsistent and non-systematic way all over the world, mainly due to the elevated cost^(5)^.

In Brazil, the first sites that implemented NHS in 1987 were Hospital São Paulo (São Paulo, SP) and Hospital Universitário de Santa Maria (Santa Maria, RS). In both places, the baby’s behavioral responses were observed in this assessment. In the following year, Hospital Israelita Albert Einstein started the first NHS program that also used the electrophysiological method Brainstem Auditory Evoked Potential (BAEP)^(6)^. In 1995, the first multi-professional recommendation on aspects related to children’s hearing health was published, as a result of a workgroup that emerged at the X International Audiology Meeting (1995)^(7)^.

On November 12, 1997, law no. 3.842/1997 was presented to make the OAE test mandatory in all public and private hospitals and maternities in the country^(8)^. The first initiatives to disseminate and support the implementation of NHS happened in 1998 with the creation of the Universal Newborn Hearing Screening Support Group^(9)^. In the following year (1999) the first national recommendation for the screening implementation was made by the Brazilian Committee on Hearing Losses in Childhood^(10)^, which followed the international principles and guidelines. The Federal Council of Speech Therapy in 2000, in the Legal Opinion no. 05/00, said that the Speech Therapist is the professional qualified to implement and execute the hearing screening programs in hospitals and maternities, and must consider the use of objective methodologies, such as recording the OAE and BAEP^(11)^.

Therefore, the Administrative Acts GM/MS no. 2073/2004^(12)^ and SAS/MS no. 587^(13)^ and 589/2004^(14)^ allowed a massive breakthrough in early intervention, establishing and regulating the National Policy for Hearing Health Care, improving the hearing health actions of the Unified Health System (SUS) and proposing the organization of a hierarchical, regionalized, and integrated network between basic, medium, and high complexity care, trying to ensure hearing diagnosis and rehabilitation. The services of Hearing Health Care in the medium complexity level should partake in the execution of NHS and hearing monitoring in neonates.

In 2010, Federal Law no. 12.303^(15)^ made screening through OAE exam mandatory in all children born in maternities and hospitals, allowing the integrality of hearing health care in childhood, following the trend of what was already happening in developed countries, such as the United States and England^(16)^. The Multi-professional Committee on Hearing Health (COMUSA)^(17)^ published an opinion to guide the actions of professionals involved in NHS programs, following two international recommendations: one from England, entitled “Guidelines for early diagnosis and treatment of infants who failed UNHS”, and one from the United States, entitled “Principles and guidelines for early detection and intervention programs of hearing impairment”, published by the *Joint Committee on Infant Hearing (*JCIH)^(16)^. The JCIH recommendations have been systematically adopted as guides for the Brazilian documentations, with scientific evidence that guides worldwide updates in the clinical practice of early identification and intervention for newborns and infants at risk of hearing loss.

In turn, the Federal Decree 7.612 of 2011^(18)^ established the National Plan of the Rights to the Person with Disability - Living without Limitation, where it established the Care Network for the Person with Disability and qualified hearing health services. Following Administrative Act no. 1.459, of June 24, 2011^(19)^, which established the network Rede Cegonha, financial resources were also allocated for the purchase of NHS equipment. The following year, the Ministry of Health published the NHS Care Guidelines^(20)^ to guide multi-professional teams for the care of auditory health in childhood, in the different points of attention of the network, with a flowchart for such. Also in 2012 were published the Hearing Health Instructions referring to Administrative Acts GM 793 of April 24, 2012^(21)^ and 835 of April 25, 2012^(22)^, with guidelines for treatment, rehabilitation and/or habilitation of people with hearing, physical, intellectual and visual impairments. These documents also regulated the operation of the Specialized Rehabilitation Centers (SRC), including standards for physical facilities, hours, and human and material resources.

The NHS aims to be a precursor strategy in the process of evaluation of infant hearing, allowing early detection of possible hearing alterations by covering all neonates, including those who do not have risk indicators for hearing impairment (RIHI)^(23)^. It is known the need for control of NHS results, monitoring, follow-up of hearing and language development, as well as diagnosis and (re)habilitation of children born throughout the national territory. Besides these, it is necessary the control quality indicators for the implementation and assessment of actions of comprehensive hearing health care in childhood.

In the literature, there is a consensus on the importance of universal screening, with coverage rates that should be equal to 95% of live births, to reach 100%^(17,20)^. However, the Brazilian rate is below this parameter. A study presented a positive evolution in the coverage of NHS in Brazil, estimating, between January 2008 and June 2015, the achievement of 31.8% of coverage, with strong inter and intraregional inequalities in the country^(24)^. In addition, there is no integration of data for the effective monitoring of neonates, from screening to their (re)habilitation when necessary.

Brazil has a continental extension, with regional, economic, social, health and cultural differences, which may interfere in the approach to the implementation of universal NHS programs in the national territory. In the current scenario, the protocols used differ. Many reasons lead professionals to adopt a specific protocol, such as the context and the constraints imposed by the socioeconomic environment^(25)^. To ensure the successful implementation of NHS, it seems necessary to assess and contemplate the possibilities of each region, and the process can be multiplied and spread in different areas of the country^(16)^. However, the final choice should consider primarily the current scientific evidence.

To understand the current scenario of NHS implementation in several Brazilian settings, almost ten years after the implementation of the law that made this procedure mandatory in the country, this study identifies the protocols used for its implementation by speech therapy professionals, the obstacles and perspectives of this performance from the perspective of these professionals, and the adjustment of NHS programs to pre-established quality indicators. This study intends to understand the current scenario of speech therapy in NHS services in Brazil, identifying its obstacles and perspectives, and checking the adequacy of the national NHS programs concerning the pre-established quality indicators.

## METHODS

The present study is an analytical observational research whose disclosure and data collection took place between August 2018 and August 2019. The study had the participation of 33 speech therapy professionals who perform NHS, who signed an Informed Consent Form made available online on Google Forms. A questionnaire (Appendix 1) presented in the same platform was used, with structured questions, outlining the personal and professional profile of the speech therapists, the characteristics of the places where they work, the professional practices, including protocols used to perform NHS and the needs identified in this context, besides the perspectives of NHS in Brazil. These questions were divided into three sections (personal identification, performance in NHS and NHS data control) and were reviewed by the authors of the study and by speech therapy specialists before the beginning of data collection.

The study sample was conceived by convenience, based on the dissemination of the research in social media platforms and email. Emails were also sent to all the Regional Councils of Speech Therapy in Brazil and universities, so that they passed on the disclosure of the research to registered professionals and/or teachers in the area. Research disclosure was also made in congresses and lectures of NHS, ministered by the first author. Inclusion criteria: being a speech therapist, working with NHS and being registered in the Speech Therapy Council. Professionals who did not complete the questionnaire (n=2) were excluded from the study.

The statistical analysis of the data collected included the descriptive analysis of variables and, as a measure of association between variables, the chi-square test was used. All analyses were performed using SPSS version 22.0. The study stems from the research project “Development of a database for integration of data from Newborn Hearing Screening in the State of Rio Grande do Sul” (Vernier LS, 2017), approved by the Research Ethics Committee of the Federal University of Health Sciences of Porto Alegre (Consubstantiated opinion no. 3.033.334).

## RESULTS

Table 1 presents the participants’ sociodemographic data and information about their professional performance. The 33 professionals who took part in the study worked in these Brazilian states: Rio Grande do Sul (n=16), Rio Grande do Norte (n=2), Rio de Janeiro (n=1), Piauí (n=1), Paraná (n=2), Pará (n=1), Minas Gerais (n=2), Mato Grosso do Sul (n=1), Goiás (n=1), São Paulo (n=4), Santa Catarina (n=1) and Roraima (n=1).

**Table 1:** Sociodemographic data and professional performance of the study participants (n=33)

Gender - n (%)	Female	n = 31 (93.94%)
	Male	n = 02 (6.06%)
Age - variation (mean)		24 - 55 years old
		(average = 35.09 years)
Time of graduation in Speech Therapy - variation (average)		02 and a half years - 22 years
		(mean=11.35 years)
Post-graduation (Lato sensu) - n (%)	Audiology	n = 12 (36.4%)
	Does not have	n = 05 (15.2%)
Post-graduation (Stricto sensu) - n (%)	Master’s	n = 12 (36.4%)
	Does not have	n = 20 (60.6%)
Brazilian region of operation - n (%)	South	n = 19 (57.58%)
	Southeast	n = 07 (21.21%)
	Northeast	n = 03 (9.09%)
	North	n = 02 (6.06%)
Type of institutional attachment - n (%)	Appointed	n = 13 (39.4%)
	Hired	n = 11 (33.3%)
	Freelance	n = 11 (33.3%)
Time of work in the institution - variation (average)		08 months - 19 years
		(mean=6.35 years)
Teaching activities at the working places - n (%)	Does not conduct any	n = 27 (81.8%)
	Holds courses	n = 04 (12.1%)
	Residency Preceptorship	n = 03 (9.1%)
Responsibility for the Audiology sector - n (%)	Yes	n = 21 (63.6%)
	No	n = 12 (36.4%)

The Speech Therapy Service was implemented between the years 1970 and 2018 in the places where the speech therapists worked, and the implementation of NHS happened between 1998 and 2019 (only two professionals did not date this event). Regarding the workload for the implementation of NHS by the professionals, a variation of 3 to 40 hours was identified (mean=14.45h). It was identified the prevalence of only one or two professionals responsible for performing NHS in each place (54.5% and 27.3%, respectively). NHS is usually performed in the outpatient clinic (54.5%), rooming-in (51.5%) and/or office (39.4%).

When asked about a possible interruption in the NHS execution in these places, 48.5% of professionals answered affirmatively. These interruptions happened between one and twelve times, and these were the most frequent reasons: need to repair the equipment (64.7%), absence of the Speech Therapy professional (35.3%), absence of equipment (23.5%) or accessory equipment (11.8%), and vacation or impossibility of work due to health reasons (5.9%).

NHS professionals also worked in other areas of Audiology (n=14), in dysphagia (n=13), orofacial motricity (n=12), language (n=7), voice (n=5) and fluency (n=4), among others. Only three professionals did not work in any other activity or area. Regarding the protocols used for NHS, none of the questions was answered unanimously by the participants, and the use was adapted according to the case and the moment, as shown in Table 2.

**Table 2:** Protocols used to perform NHS by the professionals interviewed (n=33)

	OAET (%)	BAEP (%)	Both (%)	Does not perform any, refers (%)
Protocol used to perform NHS in neonates without RIHI	90.9%	-	9.10%	-
Protocol used to performance the retest for neonates without RIHI	93.90%	-	6.10%	-
Protocol used to perform NHS in neonates with RIHI	57.60%	12.10%	21.20%	9%
Protocol used to perform the retest for neonates with RIHI	45.50%	21.20%	21.20%	12%

For the NHS execution, the speech therapists indicated to use as a guide the Guidelines of NHS Care published by the Ministry of Health (54.4%), *Joint Committee on Infant Hearing (*39.4%) and Multi-professional Committee on Hearing Health (36.4%). As for the flow records and data of NHS care, which result in quality indicators for the implementation and evaluation of actions of comprehensive hearing health care in childhood, we identified a higher control of the total number of neonates who performed NHS (87.9%) and lower control of false-positive results (21.2%) (Table 3). There is no statistically significant relationship between the choice of NHS guidelines and their respective protocols, and the clinical practice indicated by professionals, as shown in Table 4.

**Table 3:** Data on the control of information by the professionals interviewed (n=33) in the different stages of NHS

	Yes n (%)		No n (%)
Do you have a record of how many infants have been tested (NHS) by you and your team?	29 (87.9%)	4 (12.1%)	
Do you have a record of the number of infants who were referred for NHS retesting?	25 (75.8%)	8 (24.2%)	
Do you keep track of how many infants attended the retest?	24 (72.7%)	9 (27.3%)	
Do you monitor the false positive rate (NHS)?	7 (21.2%)	26 (78.8%)	
Do you perform monitoring (NHS)?	15 (46.9%)	17 (53.1%)	
Do you have a record of how many infants were referred for diagnosis?	20 (60.6%)	13 (34.9%)	
In your practice, is RIHI research conducted?	28 (84.8%)	5 (15.2%)	
Do you have a record of how many infants with RIHI have failed NHS?	16 (48.5%)	17 (51.5%)	
Do you have a record of how many infants without RIHI have failed NHS?	13 (39.4%)	20 (60.6%)	
Do you have a record of how many infants without RIHI returned for retesting?	12 (36.4%)	21 (63.6%)	
Do you have a record of how many infants with RIHI returned for retesting?	11 (33.3%)	22 (66.7)	

**Table 4:** - Relationship between the choice of NHS guidelines and the effective practice of professionals

	Perform TOAE as a test in neonates without RIHI	Perform BAEP as a test in neonates without RIHI	Perform TOAE as retest in neonates without RIHI	Perform BAEP as retest in neonates without RIHI	Perform TOAE as a test in neonates with RIHI	Perform BAEP as a test in neonates with RIHI	Perform TOAE as retest in neonates with RIHI	Perform BAEP as retest in neonates with RIHI
Joint Committee on Infant	n = 13 (100%) P=1.000	n = 2 (15.4%) P=0.547	n = 13 (100%) P=1.000	n = 0 (0.0%) P=0.261	n = 12 (92.3%) P=0.364	n = 4	n = 11 (84.6%) P=0.245	n = 5
Hearing						(30,8%) P=0.719		-38,50%
								P= 0.770
Ministry of Health of	n = 18	n = 1	n = 17 (94.4%) P=1.000	n = 3 (16.7%) P=0.233	n = 14 (77.8%) P=0.665	n = 7	n = 10 (55.6%) P=0.070	n = 9
Brazil	(100%) P=0.455	(5.6%) P=0.579				(38.9%) P=1.000		(50.0%) P=0.823
COMUSA	n = 11	n = 0	n = 12	n = 0	n = 11 (91.7%) P=0.379	n = 2	n = 10 (83.3%) P=0.259	n = 2
	(91.7%) P=0.364	(0.0%) P=0.284	(100%) P=1.000	(0.0%) P=0.284		(16.7%) P=0.133		(16.7%)
								P=0.032

Pearson’s Chi-squared Test p<0.05

When asked whether, in case of a failure in NHS, professionals already had an established place to refer and perform the audiological diagnosis, 84.8% answered positively. Regarding having a place established for referral to ISAD (Re)habilitation, Speech Therapy and otorhinolaryngological follow-up, 75.8% answered affirmatively. Regarding the referral sites, (Re)habilitation Cochlear Implant, Speech Therapy and otorhinolaryngological follow-up, 66.7% indicated having possibilities.

The factors identified by the speech therapists as reasons for not continuing the hearing evaluation by the newborns’ caregivers were: care far from the place of residence (72.7%), socioeconomic level of the caregivers (60.6%), not considering it important to carry out the evaluation (60.6%), family structure (57.6%), lack of transportation (45.5%), fear of diagnosis (33.3%), forgetting the day of the evaluation (30.3%), education of the caregivers (21.2%), and absence of a caregiver (15.2%).

Regarding data control, 81.8% of the speech therapists stated that they would use a database for NHS, taking into consideration the epidemiological control and patient referral. According to them, the information that should be in a unified database for the registration and control of NHS data include: date, place and time of NHS execution (93.9%), date, place and time of retesting (90.9%), RIHI identification (90.9%), final results (87.9%), full data of caregivers (81.8%), date, place and time of referral (78.8%), data of the responsible speech therapist (75.8%), full name of the neonate (72,7%), history of follow-ups of the newborn (66.7%), clinical history of the newborn (69.7%), maternal clinical history (57.6%), the brand of equipment used (51.5%), date of equipment calibration (42.4%), results by frequency (36.4%), paternal clinical history (21.2%), delivery data (3%) and socioeconomic data (3%).

## DISCUSSION

Although the number of respondents of the study was not significant, professionals from different regions of the country participated, in different contexts, with constant execution of NHS over the years. A study^(24)^ indicates that the coverage of NHS in Brazil has grown over time, but it is still low and has an uneven territorial distribution. This inequality can also explain this scenario of distribution of respondents.

The understanding of management, users and other health professionals about the importance of the speech therapist in the three levels of care (primary, secondary and tertiary), according to the principles of the SUS, is essential, considering the recent inclusion of speech therapy in the field of health sciences when compared to other already consolidated sciences^(26)^. The offer of speech therapy care at SUS remains scarce, but, taking into account the growing demand^(27)^, the poor distribution of such assistance persists in the country, showing that the continuous discussion about the universalization of access and the search for equity in assistance is necessary^(28)^.

Regarding the execution of NHS performance, the use of objective methodologies, such as automatic OAE and BAEP, according to pre-established criteria, allows for a safe and reliable initial assessment^(16,17,20,23)^. However, the results show a non-standardization of protocols for the execution of NHS, with predominance of the use of TOAE in all phases and cases. Therefore, the importance of implementing a universal protocol, sensitive and specific enough to avoid false-positive and false-negative results in NHS is reinforced^(29)^. In cases where only TOAE is used, it is possible to assume that there will be an increase in the total workload that the professional must dedicate to NHS, since this protocol determines a greater number of retests^(30)^. This finding probably results from the fact that, despite the guidance for the use of TOAE and BERA-A ^(17,20,23)^, Law 12.303/2010^(15)^ makes mandatory only the execution of OAE.

It is not feasible to affirm that the services that partook in this research, distributed throughout various regions of the national territory, are complying with the NHS quality indicators proposed by the JCIH^(23)^ and reaffirmed by COMUSA^(17)^ and DANHS^(20)^. After all, knowledge of the validity of procedures, the consequence of data recording, as well as false-positive rates, is fundamental to verify these indicators. The goal of NHS programs is to identify all newborns with hearing loss, with acceptable cost^(30)^. However, the data from this study indicate a difficulty in the recording and management of such information by professionals. All results from the different stages of hearing assessment of newborns should be recorded in a digital database of data management, allowing the control of information and the assessment of the quality of UNHS programs already implemented^(16)^. This reality is intended by most speech therapists, who mentioned their willingness to use such a database if it were available.

It is not possible to affirm that all newborns, even with the test and retest performed, will be diagnosed with hearing changes. Healthcare professionals still do not have enough knowledge for subsequent follow-ups, stressing the importance of the schematic sequence proposed in NHS^(23)^.

Hearing development follows gradual steps of complexity, starting already in intrauterine life. The NHS guidelines propose that all newborns have this screening and that monitoring and follow-up of hearing and language development milestones are carried out according to growth. Babies who do not pass the test should be retested and, if necessary, diagnosed and rehabilitated for hearing. Any of these steps are deeply important for the entire process; their interruption will stir important functional losses for the child’s development. In the Brazilian NHS services analyzed, the impossibility of such follow-up/monitoring was identified, according to the experience of the professionals surveyed in their workplaces. Although most infants are screened, there is no follow-up control for those who need to be retested or diagnosed, which may jeopardize the investments in the initial screening.

In this line, it was possible to examine many interruptions in the flow of NHS in the places where professionals work for different reasons. The implementation of a NHS program requires an initial investment and, with the maintenance of the equipment, the hiring of a specialized professional, the attention to the environment and the need for a follow-up network. Although the professionals have mentioned this follow-up network, problems were identified in the maintenance of equipment and hiring of replacement professionals in periods of vacation and health licenses of the respondent speech therapists.

There are no epidemiological studies on neonatal hearing loss in Brazil. Most studies in the field concern specific services. Therefore, efforts should be focused on the development of a national database, which intends to cover the information required for the care of the child at risk for hearing loss, including screening, diagnosis, and intervention when necessary^(24)^. The importation of models and data from other countries may not fit the particularities of our population and healthcare system^(24)^, although such a system seems to be seen positively and even necessary by the professionals in this study.

We hope that the knowledge provided by this study allows to reflect on the performance of the speech therapy professional in the health network, resulting in the expansion of the hiring of these professionals for Primary Care, the promotion of comprehensive care of the child population, and the increase of their access to health. It is essential to strengthening the research efforts and the scientific publications, professional investment in the three levels of care, and attention to the current legislation, because the speech therapist is part of several public policies so that the execution of NHS strengthens the good practices of hearing prevention.

We emphasize the scarcity of national literature about this study, making it difficult to compare the findings with other investigations in this area. The goal is to encourage the effective implementation of NHS guidelines in the Brazilian healthcare system, for its universalization of access and research, as well as to search for its quality indicators. This includes monitoring the entire process of infant hearing assessment follow-up, contributing to the better organization of the network of professionals and assistance to neonates.

Regarding future perspectives, we emphasize the participants’ openness to data computerization, which may require improvements in the monitoring of the NHS flow and its outcomes, allowing the implementation of Brazilian public policies regarding children’s hearing health and its improvement.

## CONCLUSION

Although NHS is guaranteed in its universality, this is not yet verifiable in services in different regions of Brazil, because the difficulties and obstacles cover the supply of professionals, including the restriction of recording relevant information for quality indicators, to the maintenance of equipment and accessories. Most of the national NHS programs presented do not meet the quality indicators proposed by the JCIH, which were indicated by COMUSA and the Ministry of Health’s Care Guidelines for NHS. Moreover, although access to actions and services should be guaranteed, there are several difficulties, often restricting the continuity of the NHS flow. Thus, knowledge of the difficulties and inequalities that affect access and effective implementation of NHS in the country allows creating further effective strategies for its universalization.

## Appendix 1 Questionnaire applied

*Mandatory

1. Email address *

Professional identification

2. What is your name? *

3. How old are you? *

4. How long have you been a graduate? *

5. Do you have a Lato Sensu Postgraduate Degree (Specialization)? If yes, in what area? Check all that apply.

Options: None; Audiology; Language; Voice; Dysphagia; Orofacial Motricity; Educational/School Speech Pathology; Occupational Speech Pathology; Neurofunctional Speech Pathology; Gerontology; Neuropsychology; Fluency; Other.

6. Do you have a Stricto Sensu post-graduate degree? If yes, which? Check only one alternative.

Options: None; Master’s degree; Doctorate; Both (master’s and doctorate)

7. In which Brazilian state are you working? Check only one option.

Options: Acre; Alagoas; Amapá; Amazonas; Bahia; Ceará; Distrito Federal; Espírito Santo; Goiás; Maranhão; Mato Grosso; Mato Grosso do Sul; Minas Gerais; Pará; Paraíba; Paraná; Pernambuco; Piauí; Rio de Janeiro; Rio Grande do Norte; Rio Grande do Sul; Rondônia; Roraima; Santa Catarina; São Paulo; Sergipe; Tocantins

8. In what city? *

9. The institution in which you work is: * Mark only one alternative.

Options: Public Body; Private Company; Public Body/Private Company; Other.

10, What is your employment relationship with the institution? Mark only one alternative.

Options: Appointed; Hired; Non-employment; Freelance; Other.

11. How long have you worked in this institution? *

12. Do you have a teaching activity at the institution? (e.g., preceptorship, internship supervision, teaching, etc.) * Check all that apply.

Options: Not practicing; Residency preceptor; Internship supervisor; Teaching courses; Research Ethics Committee; Other.

13. Are you the speech therapist in charge of the audiology service/sector? Mark only one alternative.

Options: Yes; No; Other.

Newborn Hearing Screening

14. In what year was the speech therapy service implemented in the institution? *

15. In what year was NHS implemented in the institution? *

16. What is the weekly workload that you use for the execution of NHS? *

17. How many professionals (speech therapists) work with NHS in your institution? *

18. What are the NHS locations in your institution? Check all that apply

Options: Outpatient; Rooming-in; Medical Clinic; Neonatal ICU; Office; Other.

19. Was there any interruption in the execution of the NHS in the service? Mark only one alternative.

Options: Yes; No; Don’t know.

20. If yes, how many times?

21. What is the reason? Check all that apply.

Options: Absence of Speech Therapy professional; Absence of equipment; Absence of equipment accessory; Need for equipment repair; Other.

22. Besides NHS, in what other area(s) do you work at this institution? Check all that apply.

Options: None; Audiology; Language; Voice; Dysphagia; Orofacial Motricity; Educational/School Speech Pathology; Occupational Speech Pathology; Neurofunctional Speech Pathology; Gerontology; Neuropsychology; Fluency; Other.

23. What type of examination do you use for the NHS test in neonates without RIHI (risk indicator for hearing impairment)? Mark only one alternative.

Options: OAE; BAEP; Both; Other.

24. What type of test do you use to perform the retest in NHS without RIHI (Indicator of Risk for Hearing Impairment)? Mark only one alternative.

Options: OAE; BAEP; Both; Other.

25. What type of examination do you use to perform the test in NHS with RIHI (Indicator of Risk for Hearing Impairment)? Mark only one alternative.

Options: OAE; BAEP; Both; Other.

26. What type of test do you use to perform the retest in NHS with RIHI (Indicator of Risk for Hearing Impairment)? Mark only one alternative.

Options: OAE; BAEP; Both; Other.

27. What equipment do you use to perform NHS? Check all that apply.

Options: AccuScreen; Otodynamics; Otometrics; OtoRead; Titan; Other.

28. Which guideline do you use for the execution of NHS? Check all that apply.

Options: Joint Committee on Infant Hearing (JCIH); Ministry of Health - Brazil (MS); Multi-professional Committee on Hearing Health (COMUSA); Other.

29. In the last month, how many infants were born in your institution? Mark only one alternative.

Options: Yes, I have the records (answer the next question); No records.

30. If yes, the number of infants was:

31. On average, NHS is conducted how soon after the baby is born? *

32. Do you have a record of how many infants are tested (NHS) by you and your team in the last month? Mark only one alternative.

Options: Yes, I have the records (answer the next question); No records.

33. If yes, the number of tests was:

34. How long after the test do you retest? *

35. Do you have a record of the number of infants who were referred for re-testing of NHS in the last month? Mark only one alternative.

Options: Yes, I have the records (answer the next question); No records.

36. If yes, the number of retests was:

37. Do you keep track of how many infants have attended retesting in the last month? Mark only one alternative.

Options: Yes; No.

38. If yes, how many infants attended the retest?

39. Do you control the false positive rate (NHS)? Mark only one alternative.

Options: Yes; No.

40. Do you do the monitoring (NHS)? Mark only one alternative.

Options: Yes; No.

41. If yes, how many neonates were monitored in the last month? In what situations and ages (life span) do you indicate the monitoring?

42. Do you have a record of how many infants were referred for diagnosis in the last month? Mark only one alternative.

Options: Yes, I have the records (answer the next question); No records.

43. If yes, the number of follow-ups for diagnosis was:

44. Does your practice encompass the research on RIHI (Indicator of Risk for Hearing Impairment)? * Mark only one alternative.

Options: Yes; No.

45. If yes, how many infants presented RIHI in the last month?

46. Which maternal or gestational traits that are not considered as RIHI, but that you nonetheless observe in your clinical practice, are frequently present in cases of NHS failure? *

47. Which infant traits that are not considered as RIHI, but that you nonetheless observe in your clinical practice, are frequently present in cases of NHS failure? *

48. Do you have a record of how many infants with RIHI (Indicator of Risk for Hearing Impairment) have failed NHS in the last month? Mark only one alternative.

Options: Yes, I have the records (answer the next question); No records.

49. If yes, how many infants with RIHI have failed NHS in the last month?

50. Do you have a record of how many infants without RIHI (Indicator of Risk for Hearing Impairment) have failed NHS in the last month? Mark only one alternative.

Options: Yes, I have the records (answer the next question); No records.

51. If yes, how many infants without RIHI have failed NHS in the last month?

52. Do you have a record of how many infants without RIHI returned for retesting in the last month? Mark only one alternative.

Options: Yes, I have the records (answer the next question); No records.

53. If yes, how many infants without RIHI returned for retesting in the last month?

54. Do you have a record of how many infants with RIHI returned for retesting in the last month? Mark only one alternative.

Options: No records; Yes, I have the records (answer the next question); Other.

55. If yes, the number of infants with RIHI who returned was:

56. In case of failure, do you already have an established place for referral for ISAD (Re)habilitation, Speech Therapy and otorhinolaryngological follow-up? Mark only one alternative.

Options: Yes (answer the next question); No.

57. Which location(s)?

58. In case of failure, do you already have a location established for referral to (Re)habilitation Cochlear Implant, Speech Therapy and otorhinolaryngological follow-up? Mark only one alternative.

Options: Yes (answer the next question); No.

59. Which location(s)?

60. In case of failure, do you already have an established location for referral and diagnostic execution? * Mark only one alternative.

Options: Yes (answer the next question); No.

61. Which location(s)?

62. Upon referral, you are aware of the number of neonates who: * Check all that apply.

Options: Attended the diagnosis; Began speech therapy; Sound amplification device fitting; No knowledge.

63. If you are aware, what is the number of neonate(s) in the selected items in the last month?

Newborn Hearing Screening Data

64. Do you use a database for monitoring NHS coverage? If yes, which tool do you use? * Mark all that apply.

Options: Do not use; Excel; Word; Printed spreadsheet; Software (specify under “other”); Other.

65. Would you use a database for NHS, thinking of epidemiological control and referral of patients? Mark only one alternative.

Options: Yes; No

66. Why?

67. What factors do you identify in the practice that are related to the non-continuity of the hearing evaluation? Mark all options that apply.

Options: Educational level of caregivers; Economic level of caregivers; Family structure; Absence of caregiver; Absence of transportation; Fear of diagnosis; Care far from home; Forgetting the day of care; Lack of time; Do not consider important; Other.

68. What do you think should be in a unified database for the registration and control of NHS data? Check only one alternative.

Options: Full name of the neonate; Full data of the caregivers (name, CPF, address, telephone, etc.); Data of the speech therapist; Final results; Results by frequency; Date, place and time of execution of NHS; Date, place and time of retesting appointment; Date, place and time of follow-ups; History of follow-ups; Maternal medical history; Paternal medical history; Risk indicators for hearing impairment; Clinical history of the neonate; Brand of equipment used for NHS; Date of equipment calibration; Other

69. What other information do you think could be in an NHS database?

70. In what ways could this database be useful to you? *

71. And for the institution?
