# Supplementary material for: Neonatal Hearing Screening: protocols, obstacles and perspectives of speech therapists in Brazil - 10 years of Brazilian Federal Law 12,303/2010
Source: Codas. 2022 Jan 12;34(2):e20200331. doi: 10.1590/2317-1782/20212020331 (PMC9769413; doi:10.1590/2317-1782/20212020331)
Supplement: Translation

# Translation: Triagem Auditiva Neonatal: protocolos, obstáculos e perspectivas de fonoaudiólogos no Brasil - 10 anos da Lei Federal Brasileira 12.303/2010

**Authors:** Luíza Silva Vernier, Sílvio César Cazella, Daniela Centenaro Levandowski

**Affiliations:** 1 Programa de Pós-Graduação em Ciências da Saúde, Universidade Federal de Ciências da Saúde de Porto Alegre – UFCSPA - Porto Alegre (RS), Brasil.; 2 Departamento de Informática Biomédica, Programa de Pós-Graduação em Ciências da Saúde, Programa de Pós-Graduação em Tecnologias da Informação e Gestão em Saúde, Universidade Federal de Ciências da Saúde de Porto Alegre – UFCSPA - Porto Alegre (RS), Brasil.; 3 Departamento de Psicologia, Programa de Pós-Graduação em Ciências da Saúde, Programa de Pós-Graduação em Psicologia e Saúde, Universidade Federal de Ciências da Saúde de Porto Alegre – UFCSPA - Porto Alegre (RS), Brasil.

**Keywords:** Audição, Estudos Transversais, Saúde Pública, Triagem Neonatal, Fonoaudiologia

## Abstract

**Objetivo:**

Este estudo visa conhecer o cenário atual da atuação fonoaudiológica em serviços de TAN no Brasil, identificando seus obstáculos e perspectivas, bem como verificar a adequação dos Programas de TAN nacionais aos indicadores de qualidade pré-estabelecidos.

**Método:**

Estudo observacional analítico, realizado com fonoaudiólogos no exercício da TAN no Brasil, entre agosto de 2018 e agosto de 2019, por meio de um questionário online estruturado. Foram realizadas análises descritivas e correlacionais dos dados no programa SPSS versão 22.0.

**Resultados:**

A prática efetiva da TAN entre os profissionais não foi condizente, na sua totalidade, com os protocolos oficiais. 48,5% dos fonoaudiólogos afirmaram interrupção da TAN em algum momento nos locais de trabalho, principalmente pela necessidade de reparo do equipamento (64,7%). Quanto aos registros do fluxo e dados de atendimento, que contemplam os indicadores de qualidade, houve maior controle do número total de neonatos que realizaram a TAN (87,9%) e menor controle quanto aos resultados falsos positivos (21,2%). 81,8% dos fonoaudiólogos afirmaram ter disponibilidade para utilizar um sistema para registro e controle de dados da TAN.

**Conclusão:**

Apesar de as práticas dos profissionais estarem em geral condizentes com protocolos oficiais, a não linearidade do processo e a falta de controle dos dados constituem obstáculos importantes para a qualidade dos serviços de TAN. A maioria dos programas de TAN nacionais apresentados não cumprem os indicadores de qualidade pré-estabelecidos. Considera-se que a informatização dos registros poderá beneficiar os profissionais e potencializar a implementação da TAN como prevista na legislação e políticas públicas nacionais.

## INTRODUÇÃO

### Breve histórico da triagem auditiva neonatal no Brasil

Os testes de triagem auditiva são usados há pelo menos 60 anos para detectar crianças que necessitam avaliação auditiva adicional. Na década de 40, Ewing & Ewing observaram em recém-nascidos o Reflexo Cócleo-palpebral^(1)^. Downs e Sterrit, em 1964, avaliaram as respostas comportamentais de neonatos frente a estímulo sonoro de banda estreita centrada em 3000 Hz, a 90 dBNPS. Neste mesmo estudo foi observado um elevado índice de falso negativos, e a exposição da necessidade de se elaborar um protocolo com os indicadores de risco para a deficiência auditiva^(2)^. Os programas de triagem auditiva especificamente para recém-nascidos estão sendo desenvolvidos há mais de 35 anos^(3)^. Após a comprovação das Emissões Otoacústicas (EOA), por David Kemp^(4)^, na Inglaterra, a Triagem Auditiva Neonatal (TAN) ganhou novo impulso. Contudo, aproximadamente até a década de 90, a TAN era realizada de forma inconsistente e assistemática em todo o mundo, principalmente devido ao alto custo^(5)^.

No Brasil, os primeiros locais que implementaram a TAN, em 1987, foram o Hospital São Paulo (São Paulo, SP) e o Hospital Universitário de Santa Maria (Santa Maria, RS). Em ambos os locais era utilizada a observação de respostas comportamentais do bebê nessa avaliação. No ano seguinte, o Hospital Israelita Albert Einstein iniciou o primeiro programa de TAN que utilizou também o método eletrofisiológico Potencial Evocado Auditivo de Tronco Encefálico (PEATE)^(6)^. Em 1995 foi publicada a primeira recomendação multiprofissional sobre aspectos relacionados à saúde auditiva da criança, como resultado de um grupo de trabalho que surgiu no X Encontro Internacional de Audiologia (1995)^(7)^.

No dia 12 de novembro de 1997 foi apresentado o projeto de lei 3.842/1997, para tornar obrigatória a realização do exame EOA em todos os hospitais e maternidades públicas e privadas do País^(8)^. As primeiras iniciativas para a divulgação e apoio à implantação da TAN aconteceram em 1998, com a criação do Grupo de Apoio à Triagem Auditiva Neonatal Universal^(9)^. No ano seguinte (1999) foi elaborada a primeira recomendação nacional para a implantação da triagem pelo Comitê Brasileiro sobre Perdas Auditivas na Infância^(10)^, que seguiu os princípios e diretrizes internacionais. O Conselho Federal de Fonoaudiologia, em 2000, sob Parecer nº 05/00, relatou que o Fonoaudiólogo é o profissional capacitado para a implantação e a execução de programas de triagem auditiva em hospitais e maternidades e deve considerar o uso de metodologias objetivas, como o registro das EOA e PEATE^(11)^.

Assim, as Portarias GM/MS nº 2073/2004^(12)^ e SAS/MS nº 587^(13)^ e 589/2004^(14)^ permitiram grande avanço no que diz respeito à intervenção precoce, instituindo e regulamentando a Política Nacional de Atenção à Saúde Auditiva, aprimorando as ações de saúde auditiva do Sistema Único de Saúde (SUS) e propondo a organização de uma rede hierarquizada, regionalizada e integrada entre a atenção básica, a média e a de alta complexidade, buscando garantir o diagnóstico e a reabilitação auditiva. Os serviços de Atenção à Saúde Auditiva na média complexidade deveriam atuar na realização de TAN e no monitoramento da audição em neonatos.

Em 2010, a Lei Federal nº 12.303^(15)^ tornou obrigatória a realização da triagem, por meio do exame EOA, em todas as crianças nascidas em maternidades e hospitais, possibilitando a integralidade da assistência à saúde auditiva na infância, seguindo a tendência do que já estava acontecendo em países desenvolvidos, como Estados Unidos e Inglaterra^(16)^. O Comitê Multiprofissional em Saúde Auditiva (COMUSA)^(17)^ elaborou um parecer para nortear as ações dos profissionais envolvidos nos programas de TAN, endossando duas recomendações internacionais: da Inglaterra, intitulada “Diretrizes para o diagnóstico e tratamento precoce de bebês que falharam na TANU”, e dos Estados Unidos, denominada “Princípios e diretrizes para os programas de detecção e intervenção precoces da deficiência auditiva”, publicada pelo *Joint Committee on Infant Hearing* (JCIH)^(16)^. As recomendações do JCIH têm sido adotadas sistematicamente como norteadoras para as documentações brasileiras, pois são pautadas em evidências científicas que direcionam atualizações em nível mundial na prática clínica de identificação precoce e de intervenção para recém-nascidos e bebês em risco de perda auditiva.

Por sua vez, o Decreto Federal 7.612 de 2011^(18)^ estabeleceu o Plano Nacional dos Direitos à Pessoa com Deficiência – Viver sem Limite, o qual criou a Rede de Cuidados à Pessoa com Deficiência e qualificou serviços de saúde auditiva. Pela Portaria n° 1.459, de 24 de junho de 2011^(19)^, que instituiu a Rede Cegonha, também foram fornecidos recursos financeiros para a compra de equipamentos de TAN. No ano seguinte, o Ministério da Saúde publicou as Diretrizes de Atenção da TAN^(20)^, com o objetivo de oferecer orientações às equipes multiprofissionais para o cuidado da saúde auditiva na infância, nos diferentes pontos de atenção da rede, apresentando um fluxograma para tal. Ainda em 2012 foram publicados os Instrutivos da Saúde Auditiva, referentes às Portarias GM 793, de 24 de abril de 2012^(21)^, e 835, de 25 de abril de 2012^(22)^, referenciando diretrizes para tratamento, reabilitação e/ou habilitação de pessoas com deficiência auditiva, física, intelectual e visual. Tais documentos também regulamentaram o funcionamento dos Centros Especializados em Reabilitação (CER), incluindo normas para as instalações físicas, horário de funcionamento e recursos humanos e materiais.

A TAN tem como objetivo ser uma estratégia precursora no processo de avaliação da audição infantil, permitindo a detecção precoce de possíveis alterações auditivas ao abranger todos os neonatos, inclusive os que não possuem indicadores de risco para deficiência auditiva (IRDA)^(23)^. Sabe-se da necessidade de controle dos resultados da TAN, de monitoramento, de acompanhamento do desenvolvimento da audição e da linguagem, assim como do diagnóstico e da (re)habilitação das crianças nascidas em todo o território nacional. Além destes, é necessário o controle dos indicadores de qualidade para a implementação e a avaliação das ações da atenção integral à saúde auditiva na infância.

Na literatura há consenso quanto à importância da universalidade da triagem, com índices de cobertura que devem ser iguais a 95% dos nascidos vivos, com meta de alcançar 100%^(17,20)^. Porém, o índice brasileiro está abaixo desse parâmetro. Um estudo apresentou uma evolução positiva na cobertura da TAN no Brasil, estimando, entre janeiro de 2008 e junho de 2015, o alcance de 31,8% da cobertura, ainda com fortes desigualdades inter e intrarregionais no país^(24)^. Além disso, não existe integração dos dados para o efetivo acompanhamento dos neonatos, desde a triagem até a sua (re)habilitação, quando necessário.

O Brasil tem uma extensão continental, com diversidades regionais, econômicas, sociais, sanitárias e culturais, que podem interferir na abordagem a ser utilizada na implantação dos programas de TAN universal no território nacional. No atual cenário, os protocolos utilizados diferem. Há muitas razões pelas quais os profissionais optam por adotar um protocolo específico, como o contexto e as restrições impostas pelo ambiente socioeconômico^(25)^. Assim, para o sucesso da implantação da TAN, parece ser necessário avaliar e contemplar as possibilidades de cada região, de modo que o processo possa ser multiplicado e difundido nas mais variadas áreas do país^(16)^. Entretanto, a escolha final deveria considerar prioritariamente as evidências científicas atuais.

Buscando conhecer o cenário atual de execução da TAN em diversos cenários do Brasil, transcorridos quase dez anos desde a implementação da Lei que tornou esse procedimento obrigatório no país, este estudo identifica os protocolos utilizados para a sua realização por profissionais da Fonoaudiologia, os obstáculos e perspectivas dessa atuação na visão desses profissionais e a adequação dos Programas de TAN aos indicadores de qualidade pré-estabelecidos. Este estudo visa conhecer o cenário atual da atuação fonoaudiológica em serviços de TAN no Brasil, identificando seus obstáculos e perspectivas, bem como verificar a adequação dos Programas de TAN nacionais aos indicadores de qualidade pré-estabelecidos.

## MÉTODOS

Trata-se de pesquisa observacional analítica, cuja divulgação e coleta de dados ocorreu entre agosto de 2018 e agosto de 2019. Participaram do estudo 33 profissionais fonoaudiólogos atuantes na realização da TAN, que assinaram um Termo de Consentimento Livre e Esclarecido disponibilizado online via Google Forms. Foi utilizado um questionário (Anexo 1), apresentado na mesma plataforma, com questões estruturadas, que traçavam o perfil pessoal e profissional dos fonoaudiólogos, as características dos locais de atuação, as práticas profissionais, incluindo os protocolos utilizados para a execução da TAN e as necessidades identificadas nesse contexto de atuação, e as perspectivas da TAN no Brasil. Essas questões estavam divididas em três seções (identificação pessoal, atuação na TAN e controle de dados da TAN) e foram revisadas pelos autores do estudo e por especialistas da área da Fonoaudiologia antes do início da coleta de dados.

A amostra do estudo foi constituída por conveniência, a partir da divulgação da pesquisa em redes sociais e via e-mail. Também foram enviados e-mails para todos os Conselhos Regionais de Fonoaudiologia do Brasil e universidades, para que repassassem a divulgação da pesquisa aos profissionais cadastrados e/ou professores da área. Ainda, foram realizadas divulgações da pesquisa em congressos e palestras que abordavam a TAN, ministradas pela primeira autora. Os critérios de inclusão empregados foram: ser fonoaudiólogo(a), atuar na TAN e estar registrado no Conselho de Fonoaudiologia. Foram excluídos do estudo os profissionais que não completaram o preenchimento do questionário (n=2).

A análise estatística dos dados coletados compreendeu a análise descritiva das variáveis e, para medida de associação entre variáveis, utilizou-se o teste qui-quadrado. Todas as análises foram realizadas empregando-se o SPSS versão 22.0. O estudo deriva do projeto de pesquisa “Desenvolvimento de um banco de dados para integração de dados da Triagem Auditiva Neonatal no Estado do Rio Grande do Sul” (Vernier LS, 2017), aprovado pelo Comitê de Ética em Pesquisa da Universidade Federal de Ciências da Saúde de Porto Alegre (Parecer consubstanciado número 3.033.334).

## RESULTADOS

A Tabela 1 apresenta os dados sociodemográficos dos participantes e informações sobre a sua atuação profissional. Os 33 profissionais que participaram do estudo atuavam nos seguintes estados brasileiros: Rio Grande do Sul (n=16), Rio Grande do Norte (n=2), Rio de Janeiro (n=1), Piauí (n=1), Paraná (n=2), Pará (n=1), Minas Gerais (n=2), Mato Grosso do Sul (n=1), Goiás (n=1), São Paulo (n=4), Santa Catarina (n=1) e Roraima (n=1).

**Tabela 1:** Dados sociodemográficos e de atuação profissional dos participantes do estudo (n=33).

Gênero - n (%)	Feminino	n = 31 (93,94%)
	Masculino	n = 02 (6,06%)
Idade – variação (média)		24 - 55 anos
		(média = 35,09 anos)
Tempo de formação no curso superior de Fonoaudiologia – variação (média)		02 anos e meio - 22 anos
		(média=11,35 anos)
Pós-graduação (Lato sensu) - n (%)	Audiologia	n = 12 (36,4%)
	Não possui	n = 05 (15,2%)
Pós-graduação (Stricto sensu) - n (%)	Mestrado	n = 12 (36,4%)
	Não possui	n = 20 (60,6%)
Região brasileira de atuação - n (%)	Sul	n = 19 (57,58%)
	Sudeste	n = 07 (21,21%)
	Nordeste	n = 03 (9,09%)
	Norte	n = 02 (6,06%)
	Centro-Oeste	n = 02 (6,06%)
Tipo de vínculo institucional - n (%)	Concursado	n = 13 (39,4%)
	Contratado	n = 11 (33,3%)
	Autônomo	n = 11 (33,3%)
Tempo de atuação na instituição – variação (média)		08 meses - 19 anos
		(média=6,35 anos)
Atividades de ensino nos locais de atuação - n (%)	Não exerce	n = 27 (81,8%)
	Ministra cursos	n = 04 (12,1%)
	Preceptoria de Residência	n = 03 (9,1%)
Responsabilidade pelo setor de Audiologia - n (%)	Sim	n = 21 (63,6%)
	Não	n = 12 (36,4%)

O Serviço de Fonoaudiologia foi implementado entre os anos de 1970 e 2018 nos locais de atuação atual dos fonoaudiólogos e a implementação da TAN se deu entre 1998 e 2019 (apenas dois profissionais não sabiam datar esse acontecimento). Quanto à carga horária destinada à realização da TAN pelos profissionais, observou-se uma variação de 3 a 40 horas (média=14,45h). Observou-se prevalência de somente um ou dois profissionais responsáveis pela realização da TAN em cada local (54,5% e 27,3%, respectivamente). A TAN costuma ser realizada em ambulatório (54,5%), alojamento conjunto (51,5%) e/ou consultório (39,4%).

Quando questionados se, em algum momento, houve interrupção na execução da TAN nesses locais, 48,5% dos profissionais responderam afirmativamente à questão. Tais interrupções aconteceram de uma a doze vezes, sendo os motivos mais frequentes: necessidade do reparo do equipamento (64,7%), ausência do profissional da Fonoaudiologia (35,3%), ausência de equipamento (23,5%) ou de acessório do equipamento (11,8%), e férias ou impossibilidade de trabalho por questões de saúde (5,9%).

Os profissionais que atuam na TAN também atuavam em outras áreas da Audiologia (n=14), em disfagia (n=13), motricidade orofacial (n=12), linguagem (n=7), voz (n=5) e fluência (n=4), entre outras. Somente três profissionais não trabalhavam em nenhuma outra atividade ou área. Sobre os protocolos utilizados para a realização da TAN, nenhum dos quesitos foi respondido de forma unânime pelos participantes, sendo o uso adaptado de acordo com o caso e o momento, como consta na Tabela 2.

**Tabela 2:** Protocolos utilizados para a realização da TAN pelos profissionais entrevistados (n=33)

	EOAT (%)	PEATE (%)	Ambos (%)	Não realiza, encaminha (%)
Protocolo utilizado para a realização da TAN nos neonatos sem IRDA	90,90%	-	9,10%	-
Protocolo utilizado para a realização do reteste para neonatos sem IRDA	93,90%	-	6,10%	-
Protocolo utilizado para a realização da TAN nos neonatos com IRDA	57,60%	12,10%	21,20%	9%
Protocolo utilizado para a realização do reteste para neonatos com IRDA	45,50%	21,20%	21,20%	12%

Para a realização da TAN, os fonoaudiólogos mencionaram utilizar como guia as Diretrizes de Atenção da TAN, publicadas pelo Ministério da Saúde (54,4%), do *Joint Committee on Infant Hearing* (39,4%) e do Comitê Multiprofissional de Saúde Auditiva (36,4%). Quanto aos registros do fluxo e dos dados de atendimento da TAN, que resultam em indicadores de qualidade para a implantação e avaliação das ações voltadas à atenção integral à saúde auditiva na infância, observou-se maior controle do número total de neonatos que realizaram a TAN (87,9%) e menor controle dos resultados falsos positivos (21,2%) (Tabela 3). Não há relação estatisticamente significativa entre a escolha das diretrizes da TAN e seus respectivos protocolos, e a prática clínica apontada pelos profissionais, conforme apresentado na Tabela 4.

**Tabela 3:** Dados sobre o controle de informações pelos profissionais entrevistados (n=33) nas diferentes etapas da TAN

	Sim n (%)		Não n (%)
Você tem o registro de quantos bebês realizaram teste (TAN) por você e sua equipe?	29 (87,9%)	4 (12,1%)	
Você tem o registro do número de bebês que foram encaminhados para o reteste da TAN?	25 (75,8%)	8 (24,2%)	
Você tem o controle de quantos bebês compareceram ao reteste?	24 (72,7%)	9 (27,3%)	
Você faz o controle do índice de falsos positivos (TAN)?	7 (21,2%)	26 (78,8%)	
Você realiza monitoramento (TAN)?	15 (46,9%)	17 (53,1%)	
Você tem o registro de quantos bebês foram encaminhados para diagnóstico?	20 (60,6%)	13 (34,9%)	
Na sua prática é realizada a pesquisa de IRDA?	28 (84,8%)	5 (15,2%)	
Você tem o registro de quantos bebês com IRDA falharam na TAN?	16 (48,5%)	17 (51,5%)	
Você tem o registro de quantos bebês sem IRDA falharam na TAN?	13 (39,4%)	20 (60,6%)	
Você tem o registro de quantos bebês sem IRDA retornaram para reteste?	12 (36,4%)	21 (63,6%)	
Você tem o registro de quantos bebês com IRDA retornaram para reteste?	11 (33,3%)	22 (66,7)	

**Tabela 4:** Relação entre a escolha das diretrizes da TAN e a prática efetiva dos profissionais

	Realizar EOAT como teste em neonatos sem IRDA	Realizar PEATE como teste em neonatos sem IRDA	Realizar EOAT como reteste em neonatos sem IRDA	Realizar PEATE como reteste em neonatos sem IRDA	Realizar EOAT como teste em neonatos com IRDA	Realizar PEATE como teste em neonatos com IRDA	Realizar EOAT como reteste em neonatos com IRDA	Realizar PEATE como reteste em neonatos com IRDA
Joint Committee on Infant Hearing	n = 13 (100%) P=1,000	n = 2 (15,4%) P=0,547	n = 13 (100%) P=1,000	n = 0 (0,0%) P=0,261	n = 12 (92,3%) P=0,364	n = 4 (30,8%) P=0,719	n = 11 (84,6%) P=0,245	n = 5 (38,5%) P= 0,770
Ministério da Saúde do Brasil	n = 18 (100%) P=0,455	n = 1 (5,6%) P=0,579	n = 17 (94,4%) P=1,000	n = 3 (16,7%) P=0,233	n = 14 (77,8%) P=0,665	n = 7 n(38,9%) P=1,000	n = 10 (55,6%) P=0,070	n = 9 (50,0%) P=0,823
COMUSA	n = 11 (91,7%) P=0,364	n = 0 (0,0%) P=0,284	n = 12 (100%) P=1,000	n = 0 (0,0%) P=0,284	n = 11 (91,7%) P=0,379	n = 2 (16,7%) P=0,133	n = 10 (83,3%) P=0,259	n = 2 (16,7%) P=0,032

Teste Qui Quadrado de Pearson p<0,05

Quando questionados se, em caso de falha na TAN, os profissionais já tinham um local estabelecido para o encaminhamento e a realização de diagnóstico audiológico, 84,8% responderam positivamente. Já sobre ter um local estabelecido para encaminhamento para (Re)habilitação AASI, Terapia Fonoaudiológica e seguimento otorrinolaringológico, 75,8% responderam afirmamente. Quanto aos locais para encaminhamento, (Re)habilitação Implante Coclear, Terapia Fonoaudiólogica e seguimento otorrinolaringológico, 66,7% indicaram ter possibilidades.

Os fatores que os fonoaudiólogos identificaram como motivos para a não continuidade da avaliação auditiva por parte dos responsáveis dos neonatos foram: atendimento distante da residência (72,7%), nível socioeconômico dos responsáveis (60,6%), não julgar importante a realização da avaliação (60,6%), estrutura familiar (57,6%), ausência de transporte (45,5%), receio do diagnóstico (33,3%), esquecimento do dia do atendimento (30,3%), escolaridade dos responsáveis (21,2%), e ausência de responsável (15,2%).

Visando o controle de dados, 81,8% dos fonoaudiólogos afirmaram que utilizariam um banco de dados para a TAN, pensando no controle epidemiológico e encaminhamento de pacientes. Segundo eles, as informações que deveriam constar em um banco de dados unificado para o registro e controle de dados da TAN incluem: data, local e horário de realização da TAN (93,9%), data, local e horário de realização do reteste (90,9%), identificação do IRDA (90,9%), resultados finais (87,9%), dados completos dos responsáveis (81,8%), data, local e horário do encaminhamento (78,8%), dados do fonoaudiólogo responsável (75,8%), nome completo do neonato (72,7%), histórico de encaminhamentos do neonato (66,7%), histórico clínico do neonato (69,7%), histórico clínico materno (57,6%), marca do equipamento utilizado (51,5%), data de calibração do equipamento (42,4%), resultados por frequências (36,4%), histórico clínico paterno (21,2%), dados do parto (3%) e socioeconômicos (3%).

## DISCUSSÃO

Embora o número de respondentes do estudo não tenha sido expressivo, contou-se com a participação de profissionais que atuam em regiões distintas do país, em contextos diversos, com constante implantação da TAN no decorrer dos anos. Um estudo^(24)^ aponta que a cobertura da TAN no Brasil tem crescido ao longo do tempo, mas ainda é baixa e apresenta uma distribuição desigual no território. Essa desigualdade também pode explicar esse panorama de distribuição dos respondentes.

O entendimento por parte da gestão, usuários e outros profissionais das saúde quanto à importância do fonoaudiólogo atuante nos três níveis de atenção (primário, secundário e terciário), de acordo com os princípios norteadores do SUS, é essencial, levando em conta a recente inserção da Fonoaudiologia no campo das ciências da saúde quando comparada a outras ciências já consolidadas^(26)^. A oferta dos cuidados fonoaudiológicos no SUS ainda é escassa, mas, com a crescente demanda^(27)^, persiste uma má distribuição dessa assistência no país, evidenciando a necessidade contínua de discussões sobre a universalização do acesso e a busca pela equidade na assistência^(28)^.

Particularmente no que tange à realização da TAN, a utilização de metodologias objetivas, como as EOA e o PEATE automáticos, de acordo com os critérios pré-estabelecidos, permite que se realize a avaliação inicial de forma segura e confiável^(16,17,20,23)^. Contudo, os resultados demonstram a não padronização de protocolos para a execução da TAN, com predominância da utilização das EOAT em todas as etapas e em todos os casos. Sendo assim, reforça-se a importância da implantação de um protocolo universal, que seja sensível e específico o suficiente, a fim de evitar resultados falso-positivos e falsos-negativos na TAN^(29)^. Nos casos de utilização somente de EOAT, é possível supor que haverá um aumento na carga horária total que o profissional deverá dedicar para realizar a TAN, uma vez que este protocolo determina maior número de retestes^(30)^. Esse achado provavelmente decorre do fato de que, apesar do direcionamento para a utilização das EOAT e PEATE-A^(17,20,23)^, a Lei 12.303/2010^(15)^ torna obrigatória somente a realização de EOA.

Não é possível afirmar que os serviços que participaram desta pesquisa, locados em diversas regiões do território nacional, estão cumprindo os indicadores de qualidade da TAN propostos pelo JCIH^(23)^ e reafirmados pela COMUSA^(17)^ e pela DATAN^(20)^, porque o conhecimento, consequência do registro de dados, da validade dos procedimentos, bem como das taxas de falso-positivo, é fundamental para a verificação desses indicadores. A meta dos programas de TAN é identificar todos os recém-nascidos com deficiência auditiva, com custo aceitável^(30)^. Contudo, os dados do presente estudo indicam uma dificuldade no registro e no gerenciamento dessas informações pelos profissionais. Entende-se que todos os resultados das diferentes etapas da avaliação auditiva dos recém-nascidos deveriam ser registrados em um banco de gerenciamento de dados digital, permitindo o controle das informações e, com isso, a avaliação da qualidade dos programas de TANU já implantados^(16)^. Essa realidade é desejada pela maioria dos fonoaudiólogos, que apontaram a vontade de utilizar um banco de dados dessa natureza, caso estivesse disponível.

Atenta-se ao fato de não ser possível afirmar que todos os recém nascidos, mesmo que com o teste e reteste realizados, serão diagnosticados com alteração auditiva, uma vez que ainda há falta de reconhecimento dos profissionais da área da saúde para os encaminhamentos subsequentes, reforçando a importância da sequência esquemática proposta na TAN^(23)^.

O desenvolvimento auditivo segue etapas graduais de complexidade, com início já na vida intra-uterina. As diretrizes da TAN propõem que todos os recém-nascidos façam essa triagem e que se mantenha o monitoramento e o acompanhamento dos marcos do desenvolvimento da audição e da linguagem, conforme o crescimento avança. Bebês que não passaram no teste deverão realizar o reteste e, se necessário, o diagnóstico e a reabilitação auditiva. Quaisquer dessas etapas são de grande importância para que todo o processo se complete; sua interrupção levará, consequentemente, a prejuízos funcionais importantes para o desenvolvimento da criança. No cenário dos serviços brasileiros de TAN analisados, percebeu-se a impossibilidade desse acompanhamento/monitoramento, conforme a experiência dos profissionais pesquisados nos seus locais de atuação. Com isso, embora a grande maioria dos bebês esteja sendo triada, não se tem o controle de seguimento para aqueles que precisam ser retestados ou diagnosticados, o que pode colocar a perder os investimentos na triagem inicial.

Nessa mesma direção, foi possível observar muitas interrupções no fluxo da TAN nos locais em que os profissionais atuavam, por diferentes motivos. Sabe-se que a implantação de um programa de TAN requer um investimento inicial e com a manutenção do equipamento, a contratação de profissional especializado, o cuidado com a ambiência e a necessidade de uma rede para encaminhamentos. Embora os profissionais tenham mencionado essa rede de encaminhamentos, foram encontrados problemas para a manutenção dos equipamentos e a contratação de profissionais substitutos em períodos de férias e licenças de saúde dos fonoaudiólogos respondentes.

Não existem estudos epidemiológicos sobre a perda auditiva neonatal no Brasil. A maioria dos estudos da área se referem a serviços específicos. Portanto, esforços devem ser voltados para o desenvolvimento de um banco de dados nacional, que busque contemplar as informações necessárias ao cuidado da criança com risco para deficiência auditiva, incluindo a triagem, o diagnóstico e a intervenção, quando necessário^(24)^. A importação de modelos e dados de outros países pode não atender as particularidades da nossa população e do nosso sistema de saúde^(24)^, embora um sistema dessa natureza pareça ser bem visto e até mesmo necessário pelos profissionais desse estudo.

Espera-se que o conhecimento produzido nesse estudo forneça subsídios para a reflexão sobre a atuação do profissional da Fonoaudiologia na rede de saúde, resultando na ampliação da contratação desse profissional na Atenção Básica, para a promoção da integralidade do cuidado da população infantil e ampliação do seu acesso à saúde. Mostra-se indispensável fortalecer as pesquisas e as publicações científicas, investimento profissional nos três níveis de atenção, e atenção às legislações vigentes, visto que o fonoaudiólogo é inserido em diversas políticas públicas, para que a realização da TAN efetivamente fortaleça as boas práticas de prevenção auditiva.

Reforça-se a escassez de literatura nacional sobre o tema desse estudo, o que dificulta a comparação dos achados com outras pesquisas da área. Almeja-se incentivar a implementação efetiva das diretrizes da TAN no sistema de saúde brasileiro, para a sua universalização em termos de acesso e pesquisa, bem como buscar seus indicadores de qualidade, o que inclui o monitoramento de todo o processo de acompanhamento da avaliação auditiva infantil, contribuindo, assim, para a melhor organização da rede de profissionais e da assistência aos neonatos.

Como perspectivas futuras, ressalta-se a abertura dos participantes à informatização dos dados, o que poderá implicar em melhorias no monitoramento do fluxo da TAN e nos seus desfechos, possibilitando a efetiva implantação das políticas públicas brasileiras referentes à saúde auditiva infantil e o seu aperfeiçoamento.

## CONCLUSÃO

Embora a TAN seja garantida na sua universalidade, ainda não se observa isso nos serviços de diferentes regiões do Brasil, visto que as dificuldades e obstáculos vão desde a oferta de profissionais, incluindo a restrição de registro de informações pertinentes aos indicadores de qualidade, até a manutenção de equipamentos e acessórios. Verificou-se que a maioria dos programas de TAN nacionais apresentados não cumprem os indicadores de qualidade propostos pelo JCIH, os quais foram referenciados pelo COMUSA e pelas Diretrizes de Atenção da TAN do Ministério da Saúde. Além disso, embora o acesso às ações e serviços devam ser assegurados, dificuldades estão presentes, muitas vezes restringindo a continuidade do fluxo da TAN. Assim, o conhecimento das dificuldades e desigualdades que afetam o acesso e a realização efetiva da TAN no país permite elaborar estratégias mais efetivas para a sua universalização.

## Anexo 1 Questionário aplicado

*Obrigatório

1. Endereço de e-mail *

Identificação profissional

2. Qual é o seu nome? *

3. Qual é a sua idade? *

4. Há quanto tempo está formado(a)? *

5. Você possui Pós Graduação Lato Sensu (Especialização)? Se sim, qual área? * Marque todas que se aplicam.

Opções: Nenhuma; Audiologia; Linguagem; Voz; Disfagia; Motricidade Orofacial; Fonoaudiologia Educacional/Escolar; Fonoaudiologia do Trabalho; Fonoaudiologia Neurofuncional; Gerontologia; Neuropsicologia; Fluência; Outro.

6. Você possui pós-graduação Stricto Sensu? Se sim, qual? * Marcar apenas uma alternativa.

Opções: Não possuo; Mestrado; Doutorado; Ambos (mestrado e doutorado)

7. Em que estado brasileiro você está atuando? * Marcar apenas uma alternativa.

Opções: Acre; Alagoas; Amapá; Amazonas; Bahia; Ceará; Distrito Federal; Espírito Santo; Goiás; Maranhão; Mato Grosso; Mato Grosso do Sul; Minas Gerais; Pará; Paraíba; Paraná; Pernambuco; Piauí; Rio de Janeiro; Rio Grande do Norte; Rio Grande do Sul; Rondônia; Roraima; Santa Catarina; São Paulo; Sergipe; Tocantins

8. Em que cidade? *

9. A instituição em que você trabalha é: * Marcar apenas uma alternativa.

Opções: Órgão público; Empresa Privada; Órgão público/Empresa privada; Outro.

10, Como é o seu vínculo empregatício com a instituição? * Marcar apenas uma alternativa.

Opções: Concursado; Contratado; Sem vínculo; autônomo; Outro.

11. Há quanto tempo você atua nessa instituição? *

12. Você exerce atividade de ensino dentro da instituição? (ex.: preceptoria, supervisão de estágio, docência, etc) * Marque todas que se aplicam.

Opções: Não exerço; Preceptor de residência; Supervisor de estágio; Ministrar cursos; Comitê de Ética em Pesquisa; Outro.

13. Você é o(a) fonoaudiólogo(a) responsável pelo serviço/setor de audiologia? * Marcar apenas uma alternativa.

Opções: Sim; Não; Outro.

Triagem Auditiva Neonatal

14. Em que ano o serviço de fonoaudiologia foi implantado na instituição? *

15. Em que ano a TAN foi implantada da instituição? *

16. Qual a carga horária semanal destinada por você para a realização da TAN? *

17. Quantos profissionais (fonoaudiólogos) trabalham com TAN na sua instituição? *

18. Quais são os locais de realização da TAN dentro da sua instituição? * Marque todas que se aplicam

Opções: Ambulatório; Alojamento Conjunto; Clínica Médica; UTI Neonatal; Consultório; Outro.

19. Em algum momento houve interrupção na execução da TAN no serviço? * Marcar apenas uma alternativa.

Opções: Sim; Não; Não sei.

20. Se sim, quantas vezes?

21. Qual motivo? *Marque todas que se aplicam.

Opções: Ausência de profissional da Fonoaudiologia; Ausência de equipamento; Ausência de acessório do equipamento; Necessidade de reparo do equipamento; Outro.

22. Além de realizar a TAN, em qual(is) outra(s) área(s) você atua nesta instituição? * Marque todas que se aplicam.

Opções: Nenhuma; Audiologia; Linguagem; Voz; Disfagia; Motricidade Orofacial; Fonoaudiologia Educacional/Escolar; Fonoaudiologia do Trabalho; Fonoaudiologia Neurofuncional; Gerontologia; Neuropsicologia; Fluência; Outro.

23. Que tipo de exame você utiliza para a realização do teste na TAN nos neonatos sem IRDA (indicador de risco para deficiência auditiva)? * Marcar apenas uma alternativa.

Opções: EOA; PEATE; Ambos; Outro.

24. Que tipo de exame você utiliza para a realização do reteste na TAN sem IRDA (Indicador de Risco para Deficiência Auditiva)? * Marcar apenas uma alternativa.

Opções: EOA; PEATE; Ambos; Outro.

25. Que tipo de exame você utiliza para a realização do teste na TAN com IRDA (Indicador de Risco para Deficiência Auditiva)? * Marcar apenas uma alternativa.

Opções: EOA; PEATE; Ambos; Outro.

26. Que tipo de exame você utiliza para a realização do reteste na TAN com IRDA (Indicador de Risco para Deficiência Auditiva)? * Marcar apenas uma alternativa.

Opções: EOA; PEATE; Ambos; Outro.

27. Quais equipamentos você utiliza para a realização da TAN? * Marque todas que se aplicam.

Opções: AccuScreen; Otodynamics; Otometrics; OtoRead; Titan; Outro.

28. Qual diretriz você utiliza para a realização da TAN? * Marque todas que se aplicam.

Opções: Joint Committee on Infant Hearing (JCIH); Ministério da Saúde - Brasil (MS); Comitê Multiprofissional em Saúde Auditiva (COMUSA); Outro.

29. No último mês, quantos bebês nasceram na sua instituição? * Marcar apenas uma alternativa.

Opções: Sim, tenho registro (responda a próxima pergunta); Não tenho registro.

30. Se sim, o número de bebês foi:

31. A TAN é realizada, em média, quanto tempo após o nascimento do bebê? *

32. Você tem o registro de quantos bebês realizaram teste (TAN) por você e sua equipe no último mês? * Marcar apenas uma alternativa.

Opções: Sim, tenho registro (responda a próxima pergunta); Não tenho registro.

33. Se sim, o número de testes foi de:

34. Quanto tempo após o teste você realiza o reteste? *

35. Você tem o registro do número de bebês foram encaminhados para o reteste da TAN no último mês? * Marcar apenas uma alternativa.

Opções: Sim, tenho registro (responda a próxima pergunta); Não tenho registro.

36. Se sim, o número de retestes foi de:

37. Você tem o controle de quantos bebês compareceram ao reteste no último mês? * Marcar apenas uma alternativa.

Opções: Sim; Não.

38. Se sim, quantos bebês compareceram ao reteste?

39. Você faz o controle do índice de falsos positivos (TAN)? * Marcar apenas uma alternativa.

Opções: Sim; Não.

40. Você realiza monitoramento (TAN)? * Marcar apenas uma alternativa.

Opções: Sim; Não.

41. Se sim, quantos neonatos realizaram no último mês? Em quais situações e idades (tempo de vida) você indica?

42. Você tem o registro de quantos bebês foram encaminhados para diagnóstico no último mês? * Marcar apenas uma alternativa.

Opções: Sim, tenho registro (responda a próxima pergunta); Não tenho registro.

43. Se sim, o número de encaminhamentos para diagnóstico foi de:

44. Na sua prática é realizada a pesquisa de IRDA (Indicador de Risco para Deficiência Auditiva)? * Marcar apenas uma alternativa.

Opções: Sim; Não.

45. Se sim, quantos bebês apresentaram IRDA no último mês?

46. Quais características maternas ou gestacionais que não são consideradas como IRDA, mas que, na prática clínica você observa que estão frequentemente presentes em casos de falha na TAN? *

47. Quais características do bebê que não são consideradas como IRDA, mas que, na prática clínica você observa que estão frequentemente presentes em casos de falha na TAN? *

48. Você tem o registro de quantos bebês com IRDA (Indicador de Risco para Deficiência Auditiva) falharam na TAN no último mês? * Marcar apenas uma alternativa.

Opções: Sim, tenho registro (responda a próxima pergunta); Não tenho registro.

49. Se sim, quantos bebês com IRDA falharam na TAN no último mês?

50. Você tem o registro de quantos bebês sem IRDA (Indicador de Risco para Deficiência Auditiva) falharam na TAN no último mês? * Marcar apenas uma alternativa.

Opções: Sim, tenho registro (responda a próxima pergunta); Não tenho registro.

51. Se sim, quantos bebês sem IRDA falharam na TAN no último mês?

52. Você tem o registro de quantos bebês sem IRDA retornaram para reteste no último mês? * Marcar apenas uma alternativa.

Opções: Sim, tenho registro (responda a próxima pergunta); Não tenho registro.

53. Se sim, quantos bebês sem IRDA retornaram para reteste no último mês?

54. Você tem o registro de quantos bebês com IRDA retornaram para reteste no último mês? * Marcar apenas uma alternativa.

Opções: Não tenho registros; Sim, tenho registro (responda a próxima pergunta); Outro.

55. Se sim, o número de bebês com IRDA que retornaram foi de:

56. Em caso de falha, você já tem um local estabelecido para encaminhamento para (Re)habilitação AASI, Terapia Fonoaudiológica e seguimento otorrinolaringológico? * Marcar apenas uma alternativa.

Opções: Sim (responda a próxima pergunta); Não.

57. Qual(is) local(is)?58. Em caso de falha, você já tem um local estabelecido para encaminhamento para (Re)habilitação Implante Coclear, Terapia Fonoaudiólogica e seguimento otorrinolaringológico? * Marcar apenas uma alternativa.

Opções: Sim (responda a próxima pergunta); Não.

59. Qual(is) local(is)?

60. Em caso de falha, você já tem um local estabelecido para encaminhamento e realização de diagnóstico? * Marcar apenas uma alternativa.

Opções: Sim (responda a próxima pergunta); Não.

61. Qual(is) local(is)?

62. Após encaminhamento, você tem conhecimento do número de neonatos que: * Marque todas que se aplicam.

Opções: Compareceram ao diagnóstico; Iniciaram terapia fonoaudiológica; Adaptação de aparelho de amplificação sonora; Não tenho conhecimento.

63. Se você tem conhecimento, qual(is) a(s) quantidade(s) de neonato(s) nos itens selecionados, no último mês?

Dados da Triagem Auditiva Neonatal

64. Você utiliza um banco de dados para o controle da cobertura de TAN? Se sim, qual ferramenta utiliza? * Marque todas que se aplicam.

Opções: Não utilizo; Excel; Word; Planilha impressa; Software (especifique em “outro”); Outro.

65. Você utilizaria um banco de dados para a TAN, pensando no controle epidemiológico e encaminhamento de pacientes? * Marcar apenas uma alternativa.

Opções: Sim; Não

66. Por quê?

67. Quais fatores você identifica na prática que estão relacionados com a não continuidade da avaliação auditiva? * Marque todas que se aplicam.

Opções: Escolaridade dos responsáveis; Nível econômico dos responsáveis; Estrutura familiar; Ausência de responsável; Ausência de transporte; Receio do diagnóstico; Atendimento distante de sua residência; Esquecimento do dia de atendimento; Ausência de tempo; Não julgar importante; Outro.

68. O que você acha que deveria constar em um banco de dados unificado para o registro e controle de dados da TAN? * Marcar apenas uma alternativa.

Opções: Nome completo do neonato; Dados completos dos responsáveis (nome, CPF, endereço, telefone, etc.); Dados do fonoaudiólogo; Resultados finais; Resultados por frequência; Data, local e horário de realização da TAN; Data, local e horário da marcação de reteste; Data, local e horário dos encaminhamentos; Histórico de encaminhamentos; Histórico clínico materno; Histórico clínico paterno; Indicadores de Risco para deficiência auditiva; Histórico clínico do neonato; Marca do equipamento utilizado para a TAN; Data da calibração do equipamento; Outro

69. Quais outras informações você acha que poderia constar em um banco de dados da TAN?

70. Em que aspectos esse banco de dados poderia ser útil para você? *

71. E para a instituição?

72. Sugestões ou demais comentários:
